# CHILDREN’S HEALTH: Sex-Specific Cognitive Effects of Lead

**DOI:** 10.1289/ehp.117-a393

**Published:** 2009-09

**Authors:** Julia R. Barrett

**Affiliations:** **Julia R. Barrett**, MS, ELS, a Madison, Wisconsin–based science writer and editor, has written for *EHP* since 1996. She is a member of the National Association of Science Writers and the Board of Editors in the Life Sciences

As a neurotoxicant, lead is especially harmful to the developing brain, and early exposures can irreversibly impair children’s cognitive and behavioral development. Although a blood lead level of 10 μg/dL is used as a benchmark for intervention, a growing body of research demonstrates that neurologic effects occur well below this level. Based on research published in *Early Human Development* in August 2009, boys may be even more susceptible than girls to damage related to very low-level lead exposure.

Sex-based susceptibility to low-level lead exposure was previously suspected, but this study is the first to document a statistically significant difference. “Entering into this research, we did not expect to find such a strong gender-based difference in response to very low lead levels, but this hypothesis was confirmed by a long series of analyses,” says lead author Wieslaw Jedrychowski, chair of epidemiology and preventive medicine in the College of Medicine at Jagiellonian University in Krakow.

The study population included 457 infants born in Krakow between January 2001 and February 2004. For inclusion in the study, mothers had to be nonsmokers aged 18 to 35 years with no history of chronic diseases such as diabetes or hypertension.

Upon enrolling in the study, expectant mothers completed a detailed questionnaire that covered demographic characteristics, pregnancy dates, and medical and reproductive history. Interviews during pregnancy and after birth provided information about secondhand tobacco smoke exposure during pregnancy and duration of breastfeeding.

At birth a cord blood sample was collected to measure lead concentration, and the Mental Development Index (MDI) of the Bayley Scales of Infant Development—a widely used tool for assessing mental development in young children—was administered to the women’s children at ages 12, 24, and 36 months to assess factors such as problem solving, memory, vocalization, and language. Normal MDI scores are 85 and above, whereas scores below 85 indicate delayed development.

Cord blood lead levels ranged from 0.44 to 4.60 μg/dL, with a median level of 1.21 μg/dL. Mean blood lead levels were not significantly different between boys and girls, nor were maternal education (an indicator of socioeconomic status), number of siblings, or prenatal and postnatal secondhand smoke exposure. Among boys, but not girls, cord blood lead levels were significantly associated with a lower MDI score at 36 months after controlling for confounding factors. With the median blood lead level (1.21 μg/dL) delineating low and high exposures, high exposure was associated with a 4.5-point deficit in boys’ MDI scores.

The research was very well done according to Herbert Needleman, a professor of psychiatry and pediatrics at the University of Pittsburgh School of Medicine. “Further,” he says, “I think it’s important because it concerns what other people have shown: that very small amounts of lead are neurotoxic.”

A probable explanation for the observed sex-based difference relates to males generally having fewer receptors for estrogen throughout the central nervous system than females, says Jedrychowski, who with his colleagues wrote, “The consequences of neurotoxicant exposure and the gender differences in the response to toxic exposure can partially depend on the protective effects of estrogen.”

Adds Needleman, “Boys are more sensitive to almost all [brain] insults—head injuries and things like that. The basic brain is female; masculinity is ‘tacked onto it,’ and it’s a more fragile apparatus.”

